# Improving recognition of delirium in clinical practice: a call for action

**DOI:** 10.1186/1471-2318-12-55

**Published:** 2012-09-14

**Authors:** Andrew Teodorczuk, Emma Reynish, Koen Milisen

**Affiliations:** 1 Institute for Ageing and Health, Campus for Vitality, Newcastle University, Newcastle Tyne, England, UK; 2 Education Department, North Tyneside Hospital, Northumbria Healthcare Trust, North Shields, NE29 8NH, UK; 3 Department of Geriatric Medicine, Victoria Hospital, Kirkcaldy, Fife, Scotland, UK; 4 Dementia Service Development Centre, University of Stirling, Stirling, Scotland, UK; 5 Centre for Health Services and Nursing Research, KU Leuven, Leuven, Belgium; 6 Leuven University Division of Geriatric Medicine, University Hospitals Leuven, Leuven, Belgium

**Keywords:** Delirium, Education, Training, Geriatric psychiatry

## Abstract

**Background:**

The purpose of this correspondence article is to report opinion amongst experts in the delirium field as to why, despite on-going training for all health professionals, delirium continues to be under recognised. Consensus was obtained by means of two conference workshops and an online survey of members of the European Delirium Association.

Major barriers to recognition at an individual level include ignorance about the benefit of treating delirium. At an organisational level, reflecting socio-cultural attitudes, barriers include a low strategic and financial priority and the fact that delirium is an orphan condition falling between specialties.

## Introduction

Delirium is the commonest medical complication in hospital with rates varying from 13% of young patients to 53% of older patients and up to 88% of patients with terminal cancer [1]. Delirium symptoms are also frequent in nursing homes, varying between 6.5% to over 50% of all nursing home residents depending on the characteristics of the nursing facilities [2,3]. Delirium is a source of distress for patients and families and has been shown to be associated with poorer short and long-term outcomes [4]. For instance, a recent meta-analysis of more than 700 subjects with delirium across seven studies and adjusted for the effect of age, sex, co morbid illness and pre-existing dementia showed a significantly increased post discharge mortality rate (hazard ratios, 1.95 [95% confidence interval, 1.51-2.52]), and similar increased risks for institutionalisation (odds ratio [OR], 2.41 [95% CI, 1.77-3.29]) and future dementia (OR, 12.52 [95% CI, 1.86-84.21]) [5]. Furthermore, compared to non-delirious patients, the average costs per day survived among patients with delirium until one year after discharge are more than 2 ½ times higher, leading to additional annual health care costs between $38 billion to $152 billion in the United States [6].

Recently, there has been greater interest in the management of delirium. For example in England NICE guidelines for Delirium have been published in 2010 [7]. Arguably this greater interest has stemmed from the emerging literature that delirium is preventable and to an extent treatable. For example, multifactorial interventions have been shown to effectively prevent delirium [8,9]. The evidence base for the treatment of delirium is less strong; however it is possible that this represents a lack of formal evidence as a consequence of a paucity of studies of a sufficient size [10].

Given the growing evidence base for effective prevention of delirium, it follows therefore that screening for this costly and highly prevalent disorder is essential for all staff working in the hospital and nursing homes, and a prerequisite for proper management [7,11]. A recent systematic review identified as many as 11 screening tools developed to date [12]. The authors concluded that the choice of screening instrument is determined by the amount of time available and found best evidence to support the widespread use of the Confusion Assessment Method. However, time required for rigorous training and implementation of valid administration of screening instruments in a real-world clinical setting may be high and onerous for busy clinicians [13,14].

Unfortunately, in practice there is converging evidence that delirium is poorly detected. Rates of under diagnosis vary from 33 to 72% and diagnostic errors include, among other things, misattribution to dementia or depression [15,16]. Furthermore diagnostic uncertainty is reflected by the widespread use of the unhelpful lay term “confusion”; a term which can be either a symptom or a diagnosis, as well as by the failure to consistently use standardized screening instruments in daily practice [17].

### Expert consensus

With this in mind, we report the findings of a three stage iterative process which aimed to gain consensus amongst experts in the delirium field as to why, despite on-going training and clear benefit from prevention strategies, delirium continues to be under recognised. Opinion was sought from participants at two conference workshops at the European Delirium Association (EDA) Scientific Congresses held in October 2010 (Amsterdam, Netherlands) and November 2011 (Umea, Sweden). The EDA Annual Scientific Congress is the largest meetings of professionals dedicated to advance understanding of delirium. Professionals represented were from the whole healthcare spectrum, though predominantly composed of doctors and nurses working either within the acute medical or mental health setting. In total delegates at the two workshops gave views from 20 countries worldwide.

Since this work was a consensus exercise designed to produce information to help delivery of good educational practice in relation to delirium care (rather than test hypothesises), it was exempt from ethical approval. Furthermore we specified to delegates that participation was voluntary and that reporting of consensus results assured their anonymous participation and did not permit identification of individual opinions.

The first workshop specifically aimed to explore barriers to detection and generate potential solutions to overcome these challenges. The second workshop, a year later, involved presentation of the initial workshop findings in order to determine the degree to which they resonated with a second group of experts. Lastly, the authors undertook an online survey of members of the European Delirium Association to further seek further opinions which may not have been expressed during the workshops. Eight responses from members were obtained over a month period. By this iterative process consensus was obtained on under recognition and an argument developed for a call for action.

1 In which settings is screening for delirium appropriate?

There was consensus that the population at risk in the acute hospital was entirely appropriate for screening. Specifically, there was agreement within the group that this should include acute hospital emergency admissions (with special attention for older persons), along with the inpatient critical care population (ICU/HDU). There was less agreement surrounding screening in community settings but the suggestion was made that screening may be the domain of the attending primary care doctor. Furthermore, in order to reduce hospital admissions of delirium there is also a need to recognise and prevent delirium in high risk settings such as nursing homes.

2 What are the barriers that prevent recognition of delirium?

Barriers to recognition of delirium emerged at an individual and organisational level. At both levels the lack of immediate recognisable benefit to bringing about delirium recognition was felt to be a major factor to hamper change. Despite the fact that ICU and Emergency settings had been identified as particularly important settings for detection, barriers fell more broadly to the hospital setting as a whole.

a Individual Level Barriers

A lack of education and general ignorance of delirium, in particular about the benefits of early recognition and treating delirium, emerged as a strong barrier to diagnosis. This may be due to preconceived ideas developed at undergraduate level as a consequence of a superficial teaching about delirium during medical or nursing studies. This echoes the findings of Davis and MacLullich who found that even though professionals may be aware of delirium they had a poor knowledge of diagnosis and treatment [18]. Furthermore, since there is competition of screening instruments from other domains, it was felt that delirium is not perceived as priority.

In terms of recognition a common diagnostic error identified was the misdiagnosis of delirium as dementia. Additionally, delirium is often perceived as a complication of another physical disease and thereby deemed, inappropriately, as being of secondary importance. In this situation the lack of clarity around all elements of the pathway, from assessment to diagnosis and treatment, added weight to the argument that recognition is crucial.

b Organisational and Cultural Barriers

At an organisational level poor leadership, both clinical and strategic, was felt to be an exacerbating factor. Leaders were felt to hold the view that “no-one will die of delirium”. This low strategic and financial priority to diagnosing delirium was seen as a factor preventing improvement in diagnosis.

There was considerable debate relating to the fact that delirium was not seen as belonging to a specific specialty. Consequently, it is perceived as an orphan condition managed haphazardly by a number of specialties and all too often falling between the gaps. This was underscored by the differing opinion, even among delegates, as to whether delirium should best be managed by general hospital staff or mental health professionals. This division in roles was articulated by expressions of “them and us” scenarios.

Views about delirium were felt to be compounded by cultural ageist attitudes which are prevalent in modern societies. More specifically, the lack of interest in geriatric care issues, which are perceived by many healthcare workers as unchallenging and not their responsibility, shape the identified organisational barriers. Finally, the lack of public awareness for delirium was highlighted. Without the existence of a patient association the public lobbying voice is unfortunately currently not part of the improvement process.

3 What can be done to improve delirium recognition and drive change?

The general consensus was that having identified barriers we had a responsibility to implement actions to overcome these challenges. Dividing barriers into individual and organisational was crucial in driving change as there was consensus that individual training alone without attending to organisational learning was unlikely to improve patient care.

Suggested appropriate training strategies incorporated developing an understanding of delirium, the poor outcomes, efficient and effective ways of recognition and management and crucially the patient experience. Furthermore, elements of modelling and opportunistic learning to showcase good practice were deemed essential. It was felt that educational innovations should be offered for all healthcare professionals at pre and post qualifying levels and where possible be freely accessible on line. For example, results of a study presented at the most recent EDA meeting showed a delirium e-learning program to be an effective educational approach to improve nurses’ knowledge about delirium [19,20]. Lastly, the importance of interprofessional education as a teaching method was also highlighted [21].

In order to overcome organisational barriers to good practice, communicating with healthcare managers to situate delirium on the corporate agenda was identified as an important driver for change. Further, showcasing examples of ongoing good practice and associated efficiency savings could be cited as a means of promoting organisational learning. For example, making explicit performance indicators for delirium may be used to guarantee effective enforcement of the quality of delirium care. This approach has been recently introduced in the Netherlands as a mandatory initiative in all hospitals by the National Health Care Inspectorate. Indicators include, *“registration of the proportion of patients with an elevated risk of delirium, in which a screening tool is used to confirm a diagnosis of delirium, regardless of the outcome”* (http://www.igz.nl/english/).

A need to place delirium on an equal footing to other medical disorders was highlighted. This would require a concerted public awareness and education programme in order to bring about societal change to this orphan illness. Strategies might include the use of widespread patient information leaflets explaining in lay language the importance of the delirium in the dementia population. Videos involving carers and patients could be developed and used as teaching tools to understand the patient experience. Lastly, public lectures and awareness events (such as a Delirium Fair), could help promote an understanding that the reasons why patients with dementia do badly in general hospitals are due to their delirium.

It was noted that in contrast to other common illnesses in late life there currently exist no dedicated patient groups (e.g. The Alzheimer’s Society in the UK) which could raise the profile and help educate the public and ultimately change attitudes. There was a feeling within the workshop that public awareness of delirium was potentially similar to perceptions of dementia 30 years ago. Lastly, some at the workshop argued that the time was right to change the name of the disorder from delirium to another name which could enhance the credibility and detract from ill formed ideas about delirium.

### Call for action and conclusions

Based on the consensus obtained the authors call for a concerted call for action to address difficulties with under recognition of delirium (Table 1). In particular, they advocate more strategic education at individual and organisational levels which should be undertaken in an integrated manner. Educational approaches, targeted at all healthcare professionals wherever possible, should focus on promoting awareness of delirium and clearer training in delirium prevention and treatment. As a first step we have developed an on-line freely accessible video of patient experience which can be used to aid effective educational approaches ^a^. An on-line freely accessible delirium e-learning tool (in Dutch) is also available ^b^, which increases access for learners, integrates knowledge and skill development (e.g. use of screening instruments such as Confusion Assessment Method (CAM [22] and CAM-ICU [23]) or Delirium Observation Screening Scale (DOSS [24])) and promotes active, problem-based learning.

**Table 1:** Call for action: suggested approaches at individual, organisational and societal level


**Individual level action**	Educational innovations (such as E-learning) for all healthcare professionals focused on promoting knowledge about delirium management and learning from patient experience to address attitudes towards patients with delirium	Interprofessional education (team learning) focused on prevention, early recognition and treatment of delirium
**Organisational level action**	Prioritisation of delirium on healthcare agendas of hospitals (including the Emergency Department, ICUs, and on transitions of care), nursing homes and home care organisations and linking to other agendas (e.g. dignity; patient safety)	Examination and redesign of systems in place to help facilitate delivery of effective delirium care (e.g. altering ward documentation to make use of systematic screening with standardised instruments such as Confusion Assessment Method (CAM [22] and CAM-ICU [23]) or Delirium Observation Screening Scale (DOSS [24]) more prominent)
**Societal level action**	Improve public knowledge of delirium through public awareness campaigns	Develop delirium patient support groups

At an organisational level there exists a requirement to place delirium on an equal footing to other life threatening disorders. To aid with this the European Delirium Association endeavours to contact the leads of healthcare organisations to help prioritise delirium and draw attention to the healthcare as well as economic benefits of elevating delirium higher up on the healthcare agenda.

In summary, we propose that the challenge for the modern day healthcare leaders will be to shift the focus of attention to delirium care and accordingly adapt systems and training around this preventable and treatable serious illness in the future. Only by means of such a concerted action will improvements in delirium care truly be gained.

### Endnotes

^a^http://www.europeandeliriumassociation.com/delirium-information/health-professionals/patient-experience-of-delirium-teaching-video/.

^b^http://www.deliriummodule.be.

## Competing interests

The authors declare that they have no competing interests.

## Author contributions

ER and KM conceptualized and facilitated the original workshop in 2010. AT took notes at this workshop. AT co facilitated the workshop in 2011 and the on line survey (with ER and KM). AT wrote the original draft and all authors have revised it. All authors read and approved the final manuscript.

## Pre-publication history

The pre-publication history for this paper can be accessed here:

http://www.biomedcentral.com/1471-2318/12/55/prepub
